# Protein quality control of cell stemness

**DOI:** 10.1186/s13619-020-00064-2

**Published:** 2020-11-12

**Authors:** Pengze Yan, Jie Ren, Weiqi Zhang, Jing Qu, Guang-Hui Liu

**Affiliations:** 1 State Key Laboratory of Membrane Biology, Institute of Zoology, Chinese Academy of Sciences, Beijing, 100101 China; 2 University of Chinese Academy of Sciences, Beijing, 100049 China; 3 China National Center for Bioinformation, Beijing, 100101 China; 4 CAS Key Laboratory of Genomic and Precision Medicine, Collaborative Innovation Center of Genetics and Development, Beijing Institute of Genomics, Chinese Academy of Sciences, Beijing, 100101 China; 5 Institute for Stem cell and Regeneration, Chinese Academy of Sciences, Beijing, 100101 China; 6 State Key Laboratory of Stem Cell and Reproductive Biology, Institute of Zoology, Chinese Academy of Sciences, Beijing, 100101 China; 7 Beijing Institute for Brain Disorders, Advanced Innovation Center for Human Brain Protection, National Clinical Research Center for Geriatric Disorders, Xuanwu Hospital Capital Medical University, Beijing, 100053 China

**Keywords:** Protein quality control, Stem cells, Stemness, Chaperones, Unfolded protein response, Ubiquitin-proteasome system, Autophagy

## Abstract

Protein quality control (PQC) systems play essential roles in the recognition, refolding and clearance of aberrant proteins, thus ensuring cellular protein homeostasis, or proteostasis. Especially, continued proliferation and differentiation of stem cells require a high rate of translation; therefore, accurate PQC systems are essential to maintain stem cell function. Growing evidence suggested crucial roles of PQC systems in regulating the stemness and differentiation of stem cells. This review focuses on current knowledge regarding the components of the proteostasis network in stem cells, and the importance of proteostasis in maintaining stem cell identity and regenerative functions. A complete understanding of this process might uncover potential applications in aging intervention and aging-related diseases.

## Background

Stem cells serve as the origin of a multicellular organism. They can divide to give rise to daughter cells that remain as stem cells or become differentiated with a specific function. The multi-differentiation potential gives stem cells unparalleled advantages in regenerative medicine. Originally, stem cells can be categorized into two main groups: embryonic stem cells (ESCs) and adult stem cells (ASCs). Yet with the development of reprogramming technologies, somatic cells can also be reprogrammed into ESC-like cells, termed as induced pluripotent stem cells (iPSCs). Collectively, ESCs and iPSCs are referred to as pluripotent stem cells (PSCs) because of their high capacity for self-renewal and their ability for multipotent differentiation, offering far-reaching potential in disease modeling and transplant therapies (Evans and Kaufman, 1981; Shu et al., 2013; Yamanaka, 2009). On the other hand, ASCs are undifferentiated cells distributed throughout the body, and have the ability to differentiate into several restricted cell types and to participate in tissue regeneration (Passier and Mummery, 2003; Wagers and Weissman, 2004). Due to their lower immunogenicity and higher safety profile, certain ASCs (eg. MSCs) are recognized as the most promising source for cell therapy (Kode et al., 2009; Pessina and Gribaldo, 2006).

Researches focused on stem cells have attracted much attention in recent years, with a particular focus on the transcription factor networks that regulate their stemness and differentiation (Avilion et al., 2003; Chambers et al., 2003; Cui et al., 2018; Nichols et al., 1998; Wang et al., 2019b). Other aspects of regulation, such as miRNAs and epigenetic modifications, have also been studied extensively (Atlasi and Stunnenberg, 2017; Avgustinova and Benitah, 2016; Croce and Calin, 2005; Foshay and Gallicano, 2009; Hsieh and Gage, 2004; Martinez and Gregory, 2010; Meissner, 2010; Shenoy and Blelloch, 2014; Wang et al., 2007; Wu and Sun, 2006; Yu et al., 2012). Although there has been less focus on posttranslational mechanisms of regulation, recent studies indicate that there is a close connection between protein quality control (PQC) and stem cell function (Assou et al., 2009; Bradley et al., 2012; Buckley et al., 2012; Fernandes et al., 2019; García-Prat et al., 2017; Geng et al., 2015; Heijmans et al., 2013; Hernebring et al., 2013; Jang et al., 2014; Kapetanou et al., 2017; Noormohammadi et al., 2018; Saretzki et al., 2004; Schroter and Adjaye, 2014; Vilchez et al., 2013).

A single cell contains billions of proteins with a total concentration ranging from 50 to 300 mg/ml (Asherie, 2004; Finka and Goloubinoff, 2013). Genetic mutation, transcriptional or translational errors, protein misfolding and aggregation can all lead to the generation of aberrant proteins (Goldberg, 2003; San Jose et al., 2020; Sigurdsson and Miharada, 2018). These aberrant proteins may form toxic aggregates and cause deleterious effects on cell function and viability, which may ultimately lead to human diseases (Balch et al., 2008; Bennett et al., 2005; Gidalevitz et al., 2011; Koyuncu et al., 2018; Powers et al., 2009; San Jose et al., 2020). In addition, accumulation of aberrant proteins in stem cells may also contribute to the aging process (Kapetanou et al., 2017; Vilchez et al., 2014b; Wang et al., 2018b).

**Fig. 1:**
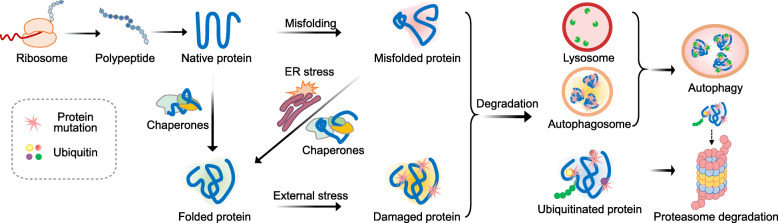
The Pathways of Protein Quality Control in Stem Cells. The four main pathways of protein quality control in stem cells are depicted here. Chaperones can facilitate the folding of polypeptides into the right structure. The unfolded protein response (UPR) is activated by misfolded proteins to aid refolding and to maintain protein homeostasis. Misfolded and damaged proteins can be degraded through the ubiquitin-proteasome system (UPS) or autophagy

To prevent aberrant proteins from accumulating, cells have evolved an elaborate network of PQC systems to recognize abnormal proteins and to facilitate their refolding or degradation (Fig. 1) (Chen et al., 2011; Goldberg, 2003; Leeman et al., 2018; Revuelta and Matheu, 2017; Richter et al., 2010). PQC systems operate since the beginning of polypeptide synthesis to avoid creating aberrant proteins, by altering the rate of translation that is dependent on the organization of ribosomes (Gingold and Pilpel, 2011; Wolff et al., 2014). There are also several pathways that ensure proteostasis after protein synthesis. Under stress conditions, the accumulation of misfolded and unfolded proteins in the cytoplasm, endoplasmic reticulum (ER), and mitochondria triggers the unfolded protein response (UPR). If aberrant proteins cannot be rescued by chaperones and the UPR pathway, they will be degraded via the ubiquitin-proteasome system (UPS) or autophagy pathways for the cell to regain protein homeostasis (Vilchez et al., 2014b). A study of the proteomic features of human embryonic stem cells (hESCs) during self-renewal stage, classified about 60 proteins as the most abundant proteins. Most of these are chaperones and UPS components (Baharvand et al., 2006), indicating that PQC systems are of great importance in maintaining stem cell pluripotency.

In this review, we highlight the importance of proteostasis in stem cells with recent advances that revealed central mechanisms by which the proteostasis network regulates stem cell function. A thorough understanding of these mechanisms will be crucial for harnessing the therapeutic potentials of stem cells and for maximizing their utility as models to understand development and diseases.

## Pathways controlling protein folding in stem cells

Proteostasis has been recognized as the cornerstone of stem cell homeostasis. The regulation of protein folding and recognition of abnormal products involve multiple components, which mainly belong to the chaperone family and the UPR pathway (García-Prat et al., 2017). In the following subsections, we discuss the critical roles of molecular chaperones and UPR in regulating cell stemness.

### Chaperones

Chaperones, which themselves are under precise regulation in the cell, are initially considered as protein folding assistants (Fink, 1999). Later, some chaperones are also found to involve in signaling the ER stress response, delivering misfolded proteins for degradation, and breaking up protein aggregates (Jarosz et al., 2010; Mcclellan et al., 2005; Taipale et al., 2010; Trepel et al., 2010). There are several different groups of chaperones, whose nomenclature is typically based on their molecular weight and stimuli in the original studies. The first group consists of high-molecular weight heat shock proteins (HSPs), including the HSP110, HSP90, HSP70, and HSP60 families. The second group of HSPs, induced by glucose starvation, includes glucose-regulated proteins (GRPs) 75 (HSPA9), 78 (HSPA5), and 94 (HSP90B1). The third group of HSPs, mainly located in the extracellular matrix, includes HSP40, HSP47, and HSP56 (Boudesco et al., 2018; Jee, 2016; Michels et al., 1997; Sterrenberg et al., 2011). Small HSPs including HSPB1-B10, whose molecular weights range from 12 to 30 kDa, are categorized as the fourth group (Fan and Kranias, 2011; Kappe et al., 2003; Sciandra and Subjeck, 1983). By analyzing proteomic profiles of human cells, numerous chaperones and co-chaperones have been identified as potential regulators that help to delay aging and aging-related diseases (Baharvand et al., 2006; Brehme et al., 2014).

**Fig. 2:**
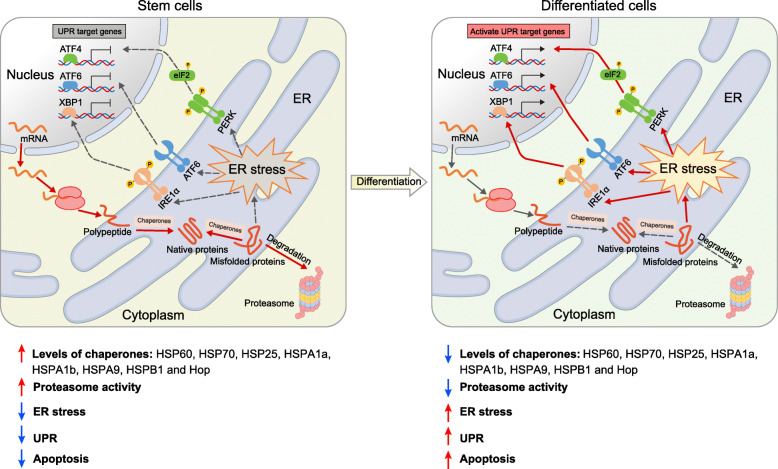
Differences of Unfolded Protein Response (UPR) Networks in Stem Cells and in differentiated Cells. Molecular chaperones facilitate the folding of nascent polypeptides into native protein and the refolding of misfolded proteins as well. If refolding fails, the chaperones deliver misfolded proteins for degradation. High levels of chaperones are present in stem cells, suggesting that stem cells have a greater capacity to assure the proper folding of proteins (Baharvand et al., 2008; Battersby et al., 2007; Saretzki et al., 2004). Similarly, the proteasome activity is also activated in stem cells, regulating the levels of key transcriptional factors and degrading misfolded proteins (Vilchez et al., 2012). Consequently, ER stress, UPR, and apoptosis are kept at a lower level in stem cells. In contrast, during differentiation, the levels of chaperones decrease, whereas ER stress increases (DeLany et al., 2005). The three signal transducers (PERK, IRE1α, and ATF6) of UPR are activated upon the increase of ER stress at the transition from stem cells to mature cell types (Hetz, 2012; Sugiura et al., 2009), suggesting their potential as markers for differentiation (Heijmans et al., 2013)

Levels of HSPs are higher in stem cells than in differentiated cells. For instance, HSPs such as HSPA1a, HSPA1b, HSPA9, and HSPB1 are highly expressed in both human and mouse embryonic stem cells (mESCs), while such high levels of expression diminish during differentiation (Baharvand et al., 2006; Battersby et al., 2007; Saretzki et al., 2004; Saretzki et al., 2008). Joint analysis of expression profiles of chaperones in human ESCs, mesenchymal stem cells (MSCs), and neural stem cells (NSCs) showed that high levels of HSPA5, HSPA8, and Stip1 expression are shared by these different types of stem cells (Baharvand et al., 2007). hESCs show a unique chaperone expression signature of HSPA4, HSPB1, and HSPCb (Baharvand et al., 2007). On the other hand, changes in the expression of certain HSPs may serve as differentiation markers of ESCs (Fan, 2012). For example, expression of HSP60, HSP70, HSP25 and co-chaperone Hop were greatly reduced during mESC differentiation (Baharvand et al., 2008; Baharvand et al., 2007; Battersby et al., 2007). Similarly, expression of HSPB1, HSPB5, and HSP60 decreases during differentiation of human adipose-derived ASCs (Delany et al., 2005). Yet, certain HSPs, such as HSPB1, do not decrease during hESC differentiation, but decrease during mESC differentiation (Saretzki et al., 2008), indicating differences in the proteostasis network between hESCs and mESCs. Nonetheless, high levels of chaperones may capacitate stem cells with greater abilities to cope with aberrant proteins and to maintain cellular homeostasis than differentiated cells. The main differences in the levels of chaperones between stem cells and differentiated cells are summarized here in Fig. 2.

In addition to the aforementioned correlative links, causal relationships have also been established between chaperones and stemness. For example, suppressing HSP90 expression contributed to mESC differentiation, at least partially because HSP90 protects Oct4 and Nanog from degradation by the proteasome (Bradley et al., 2012). *Hsp90β* knockout mice are embryonically lethal (Voss et al., 2000). The expression of HSP70 and HSP90 promotes survival of bone marrow MSCs after heat shock treatment (Wang et al., 2019a). HSP90 plays an important role in controlling the formation of hepatic progenitor cells by directly interacting with HNF4A protein, an essential transcription factor for hepatic progenitor specification from hPSCs (Jing et al., 2017). In addition, the absence of HSP60 is associated with the silencing of Oct4, and its deficiency can inhibit the proliferation and self-renewal of mESCs, and promote apoptosis as well (Seo et al., 2018).

Besides individual chaperone proteins, hPSCs also exhibit enhanced assembly of the TRiC/CCT complex, a chaperonin that promotes the folding of roughly 10% of the whole proteome and reduces toxic protein aggregation (Noormohammadi et al., 2016). CCT8, one subunit of the TRiC/CCT complex, has been identified as a key promoter of its assembly and ectopic expression of CCT8 is also sufficient to increase its assembly (Noormohammadi et al., 2016). On the contrary, during the differentiation of neural stem and progenitor cells (NSPCs), the level of TRiC/CCT complex is reduced, while small heat shock proteins are induced, thus promoting the sequestration of misfolded protein into protective inclusions and maintaining proteostasis (Vonk et al., 2020). A thorough investigation of chaperone networks in stem cell maintenance and differentiation is needed to aid our understanding of its vital role in enhancing cellular function.

### Unfolded protein response

The endoplasmic reticulum (ER) is a central cellular organelle in proteostasis. It is involved in the synthesis, modification, and delivery of proteins to their target sites in the secretory pathway and the extracellular space (Schroder and Kaufman, 2005). Under ER stress conditions, the ER unfolded protein response (UPR^ER^) is activated to cope with misfolded proteins, either facilitating their proper re-folding or delivering them for degradation via the proteasome or autophagy pathways (Araki and Nagata, 2011). Growing evidence has revealed the significance of UPR in the pathogenesis of diseases, such as cancer, metabolic syndromes and aging-related diseases (Hetz et al., 2020; Huang et al., 2019; Martínez et al., 2018; Urra et al., 2016; Wang et al., 2018a).

In the ER homeostasis, GRP78 (also named as BiP) is a central regulator, as it plays a vital role in protein folding, ER calcium binding, and regulating the activities of transmembrane ER stress sensors. Consistently, Grp78 homozygous knockout mouse embryos failed to hatch from zona pellucida, and exhibited proliferation defects and extremely high levels of apoptosis in the inner cell mass, demonstrating that Grp78 is crucial for embryonic cell growth and pluripotent cell survival (Luo et al., 2006). The signals of protein folding status are transduced to the cytosol and nucleus through activation of three different ER transmembrane proteins: ATF6 (activated transcription factor 6), PERK (double stranded RNA activated protein kinase-like ER kinase), and IRE1α (inositol-requiring transmembrane kinase and endonuclease) (Hetz, 2012). Clear proof for the impact of the UPR in ESC differentiation comes with activation of ATF6 by Dickkopf homolog 3, which promotes the differentiation of ESCs into smooth muscle cells (Wang et al., 2015).

Besides ESCs, the UPR pathway also regulates the self-renewal and differentiation of ASCs. For example, overexpressing the co-chaperone ERDJ4 (also named as DNAJB9) enhances ER protein folding, thereby increasing the repopulation capacity of hematopoietic stem cells (HSCs) in xenograft assays, connecting the UPR to the maintenance of HSC properties (van Galen et al., 2014). In human iPSC-derived cardiomyocytes, PAK2 (p21-activated kinase 2) activation can enhance ER function, reduce cell apoptosis, and protect from heart failure through the IRE1α/XBP1 (X-box binding protein 1)-dependent pathway (Binder et al., 2019). On the contrary, hematopoietic stem and progenitor cells (HSPCs) with HIF-2α knockout exhibit high levels of reactive oxygen species (ROS), which subsequently induces ER stress and apoptosis via activation of the UPR pathway (Rouault-Pierre et al., 2013). Similarly, inactivation of ATF6 impairs the ER tubular network of human MSCs and eventually leads to cellular senescence (Wang et al., 2018b). Likewise, human HSCs show a proapoptotic phenotype to prevent the proliferation of damaged stem cells after activation of the PERK branch of the UPR pathway during ER stress, as damaged HSCs are rapidly cleared whereas closely related progenitors are spared (van Galen et al., 2014). The PERK branch of the UPR has also been found to regulate the homeostasis of skeletal muscle stem cells (also known as satellite cells) during regenerative myogenesis and is crucial for their survival after activation from quiescence (Xiong et al., 2017). On the other hand, during the differentiation of rat bone marrow stromal cells and mESCs into neurons, three branches of the UPR are activated, accompanied by the expression of neuronal markers (Cho et al., 2009). ER stress during the transition from stem cells to differentiated cells activates UPR, and multipotency is lost in a PERK-eIF2-dependent manner after ER stress (Heijmans et al., 2013).

In addition to UPR^ER^, the mitochondrial unfolded protein response (UPR^MT^) also plays an important role in the maintenance of stem cell characteristics and homeostasis (Mohrin et al., 2018; Shen et al., 2020). SIRT7 inactivation can increase mitochondrial protein folding stress, reduce the regeneration ability of HSCs, and ultimately lead to aging (Mohrin et al., 2015). During stem cell reprogramming, UPR^ER^ and UPR^MT^ are both activated to ensure proteostasis, and the transient activation of UPR^ER^ is an important step in the process of reprogramming (Simic et al., 2019).

## Pathways of aberrant protein degradation in stem cells

With the decline of chaperone activity, aberrant proteins accumulate and are detrimental to stem cell function and homeostasis. Therefore, aberrant proteins are constantly removed by the UPS or through autophagy (Tanaka and Matsuda, 2014). Below we discuss recent evidence of the importance of the UPS and autophagy pathways in stem cells.

### Ubiquitin-proteasome system (UPS)

In the UPS, damaged proteins are first tagged with multiple ubiquitin molecules and then recognized and proteolyzed by the proteasome (Glickman and Ciechanover, 2002; Mukhopadhyay and Riezman, 2007; Seifert and Kruger, 2008). Active proteasomes are mainly constituted by the 20S core particle, which is the catalytic center, and the 19S regulatory particle, which identifies the polyubiquitylated substrate and unfolds it for translocation into the 20S proteolytic core (Finley, 2009; Vilchez et al., 2014a). Thus, damaged proteins are digested to short peptides (Vilchez et al., 2014b).

The UPS is involved in the maintenance of pluripotency in ESCs by both promoting the degradation of differentiation-associated proteins and maintaining the homeostasis of pluripotency-associated proteins (Fig. 1) (Assou et al., 2009; Buckley et al., 2012; Cho et al., 2014; Hernebring et al., 2013; Jang et al., 2014; Vilchez et al., 2013; Vilchez et al., 2012). In general, proteasome activity is considerably higher in pluripotent cells than in differentiated cells (Vilchez et al., 2012). For example, both hESCs and iPSCs exhibit high proteasome activity, which is correlated with increased levels of the 19S proteasome subunit PSMD11 (Vilchez et al., 2012). And differentiation of hESCs to neural progenitor cells and to mature neurons is accompanied by downregulation of PSMD11 (Vilchez et al., 2012). Consequently, ubiquitin ligases, such as LIN41, UBR5 and USP21, are also regarded as important regulators in maintaining pluripotency and reprogramming of PSCs (Koyuncu et al., 2018; Liu et al., 2016b; Nguyen et al., 2017). When stem cells are treated with MG132, a proteasome inhibitor, the levels of pluripotency markers decrease and the levels of specific germ-layer markers (*FGF5* and *GATA4*) increase (Assou et al., 2009; Vilchez et al., 2012). Transcription factors can also function through proteasome to control stem cell fate decision. For example, nuclear factor erythroid 2-like 2 (Nrf2) activates the expression of proteasome maturation protein (POMP), which in turn participates in hESC pluripotency and somatic cell reprogramming (Jang et al., 2014). Other research revealed that the proteasome could prevent transcription factors from binding to tissue-specific gene regions in ESCs, so that the differentiation-associated genes are restricted at the pluripotent cell stage (Szutorisz et al., 2006).

There is also growing evidence of the importance of the UPS in ASCs. Studies revealed that F box E3 ubiquitin ligase (FBXW7) plays a crucial role in the self-renewal and differentiation of HSCs and NSCs (Matsumoto et al., 2011; Matsuoka et al., 2008; Reavie et al., 2010; Saez et al., 2018; Thompson et al., 2008). Furthermore, a combination of RNAi screening and shotgun proteomic analysis characterizes the opposing effects between deubiquitinating enzyme PSMD14 and the E3 ligase FBXW7 in maintaining cellular pluripotency (Buckley et al., 2012). In satellite cells, deficiency of an essential proteasomal component Rpt3 can reduce the proteasome activity, impair cell proliferation, and eventually lead to cell-cycle arrest (Kitajima et al., 2018). Furthermore, proteasomes also prevent the binding of certain transcription factor and RNA polymerase Pol II to tissue-specific genes in ESCs, which could contribute to cell fate determination (Szutorisz et al., 2006). In addition, vimentin, a key regulator of cellular protein stability, can recruit proteasomes to aggresomes to remove aggregates in NSCs, and to facilitate the activation of quiescent NSCs (Maybury-Lewis and Webb, 2020; Morrow et al., 2020).

Taken together, the UPS is essential for maintaining stemness in both PSCs and ASCs, and dysfunction of the UPS may contribute to perturbed stem cell function and fate control. The main differences between UPS-related cellular reactions in stem cells and those in differentiated cells were summarized in Fig. 2. Furthermore, ER-associated degradation (ERAD) is yet another quality control mechanism that allows for the proteasomal degradation of misfolded proteins in the ER (Carvalho et al., 2006; Hampton et al., 1996). However, the function of ERAD in stem cells has not been well studied. Recently, a bioRxiv preprint revealed the essential role of ERAD in preserving the function of quiescent HSCs (Liu et al., 2019). Deficiency of an ERAD associated gene, *Sel1L*, reduced self-renewal and resulted in HSC depletion (Liu et al., 2019).

### Autophagy

Misfolded and aggregated proteins can also be degraded by a separate autophagy-mediated pathway. Autophagy is a highly conserved intracellular process, in which damaged or unwanted proteins, cytosolic fractions, and organelles are degraded by the lysosome (He and Klionsky, 2009). It is characterized by the engulfment of the targeted cytoplasmic components with a double-membrane vesicle, forming the autophagosome. The autophagosome then fuses with the lysosome, where the encapsulated contents are released and degraded by lysosomal enzymes. In this process, the released nutrients, i.e. peptides, free amino acids, and fatty acids, can be recycled for ATP generation or protein synthesis (Mizushima, 2007). Intrinsic and extrinsic stress conditions, such as ER stress, ROS, hypoxia, starvation, and bacterial infections, are well-characterized inducers of autophagy (Chen et al., 2009; Singh and Cuervo, 2011; Yorimitsu and Klionsky, 2007).

Despite extensive research on autophagy in somatic cell physiology, relatively little is known about the roles of autophagy in stem cell biology. Recent studies report that autophagy is crucial for ESCs and for various ASCs, i.e. HSCs, MSCs, NSCs, and gut stem cells, although with different requirements for its activity (He et al., 2020; Liu et al., 2017; Mizushima et al., 2001; Mortensen et al., 2011; Peng et al., 2017; Sanchez et al., 2011).

In contrast to the high levels of proteasome activity observed during self-renewal, high levels of autophagy activity was exhibited during early differentiation of ESCs and NSCs (Tra et al., 2011; Vazquez et al., 2012). For example, deficiency of *Apg5* in mESCs causes defects in autophagosome formation and consequent accumulation of proteins in the cytoplasm (Mizushima et al., 2001). Mutant mice lacking *beclin-1*, a mammalian ortholog of the yeast autophagy-related gene 6, die early in embryogenesis, and the autophagic response in mESCs is significantly altered as a result of the *beclin-1* deficiency (Yue et al., 2003). Additionally, immunogold-electron microscopy directly confirmed the localization of OCT4 molecules within autophagosomes, and inhibiting autophagy increases the accumulation of pluripotency-associated proteins in hESCs (Cho et al., 2014). In dormant NSCs, lysosomal pathways are activated to clear protein aggregates, thus restoring them to the young state (Leeman et al., 2018). Inhibition of this pathway has been related to some neurodegenerative disorders, cancers, and aging (Chen et al., 2011; Levine and Kroemer, 2008; Rubinsztein et al., 2011). Furthermore, increasing the expression of several autophagy-related genes promotes the differentiation of NSCs into neurons (Vazquez et al., 2012). NSCs with heterozygous deficiency of *Ambra1*, an autophagy gene, are impaired for neuronal generation (Vazquez et al., 2012).

Different from ESC sand NSCs, in which autophagy activity is required during differentiation, autophagy activity is decreased during the differentiation of MSCs, HSCs, dermal stem cells, and epiblast stem cells (Mortensen et al., 2011; Nuschke et al., 2014; Oliver et al., 2012; Pan et al., 2013; Salemi et al., 2012). Although hMSCs exhibit high levels of intrinsic autophagy during self-renewal, upon differentiation into osteoblasts or neurons, autophagy is attenuated to a relatively low level (Oliver et al., 2012). Similarly, upon the induction of osteogenic and adipogenic differentiation, the autophagosome marker LC3 II is lost in the early stage of differentiation (Nuschke et al., 2014). On the other hand, slightly increasing autophagy activity can protect MSCs from hypoxia- or ischemia-induced injury (Hu et al., 2019). Similarly, microRNA (miR)-142-5p was found at a high level in MSCs derived from bone marrow of aged mice and can cause ROS accumulation through the disruption of selective autophagy for peroxisomes (pexophagy). Thus, endothelial PAS domain protein 1 (EPAS1) targeted by miR-142-5p has been identified as a regulatory protein of pexophagy (Houri et al., 2020). Under starvation conditions or rapamycin treatment, autophagy is activated to cope with DNA damage induced by oxidative stress and to maintain MSC pluripotency. When the activity of autophagy is inhibited, the stemness of MSC is lost (Hou et al., 2013). Moreover, impaired autophagy activity in HSCs caused by knocking out either autophagy gene *Atg7* or *Fip200* in the hematopoietic system results in the loss of normal HSC function and death of the mice, suggesting that both autophagy genes are necessary for adult HSC maintenance (Liu et al., 2010; Mortensen et al., 2011). Maintaining a high basal autophagy flux can attenuate proteotoxicity in quiescent satellite cells (García-Prat et al., 2016). While in dormant breast cancer stem cells, targeted removal of *Atg3* or *Atg7* can inactivate autophagy and restore the expression of 6-photofructo-2-kinase/frutose-2,6-biphosphatase 3, which can in turn restart cell proliferation (Flynn et al., 2019). Blocking the expression of ATG5 or 3-methyladenine (3-MA) with shRNAs, thereby inhibiting autophagy, impairs the self-renewal capacity in epidermal stem cells, dermal stem cells, and HSCs. *Atg5* plays a key role in the maintenance of HSCs, and the reconstitution ability of *Atg5*-deficient HSCs in bone marrow of chimeric mice is significantly impaired (Jung et al., 2019). Inhibiting the sphingolipid enzyme DEGS1 can induce autophagy to maintain functional HSCs (Xie et al., 2019). Autophagy-related genes such as *Atg5*, *Atg7*, and *Atg12* can mediate the self-renewal, differentiation, and regeneration of the muscle and hematopoietic system, and the overexpression of *Atg7* can rejuvenate aged satellite cells and HSCs and restore their regeneration ability (García-Prat et al., 2016; Ho et al., 2017). Moreover, the transcription factor FOXO3 is involved in autophagy induction of NSPCs and functions through autophagy-dependent pathways in NSPC maintenance (Audesse et al., 2019). These findings strongly suggested that autophagy participates in maintaining the stemness and homeostasis of various adult stem cells.

Likewise, autophagy is essential for iPSC reprogramming. A distinguishing feature of iPSCs is that the existing number and mass of mitochondria in the somatic cell origins are strikingly diminished during reprogramming. Hence, their metabolic pattern is switched from oxidative phosphorylation to glycolysis and this is considered as an important mechanism in iPSC reprogramming. ATG3-dependent autophagy can act as an executor for mitochondrial clearance during somatic cell reprogramming (Liu et al., 2016a). Even though it is debatable whether or not the ATG5-dependent canonical autophagy is necessary for reprogramming, the two research groups agreed on that autophagy is indispensable for iPSC reprogramming (Ma et al., 2015; Wang et al., 2013). Furthermore, during the reprogramming process induced by the four Yamanaka factors, Oct4, Sox2, Klf4, and c-Myc, the expression level of mTORC1 is downregulated and autophagy-related genes are induced (Wang et al., 2013; Wu et al., 2015). However, there is also some debates about how these phenomena are triggered by the four reprogramming factors. Introducing these factors into reprogramming cells individually showed that only ectopic expression of Sox2 downregulates mTOR expression and facilitates the induction of autophagy (Wang et al., 2013). By contrast, another study suggested that the four factors repress mTORC1 collaboratively, while only Klf4 and c-Myc promote the induction of autophagy-related genes, and Sox2 and Oct4 inhibit their expression (Wu et al., 2015). In summary, the role of autophagy is crucial for successful reprogramming, but the underpinning mechanisms need to be further studied.

## Conclusions and perspectives

Previous studies have revealed that genomic and epigenetic stability is essential for stem cell identity. Yet recent years have seen increasing evidence that supported a pivotal role for proteostasis in regulating pluripotency and differentiation of stem cells as well. Under physiological conditions, the proteostasis network is equipped with high versatility in response to distinct stimuli (Balch et al., 2008; Labbadia and Morimoto, 2015; Powers et al., 2009). During the differentiation of hESCs, both proteasome and autophagy are activated to prevent the delivery of damaged proteins to their self-renewed and differentiated counterparts. Unlike somatic cells, stem cells exhibit a highly regulated network for protein homeostasis as related to their biological characteristics (Lee et al., 2017). Even though transcriptional factors and epigenetic regulators have been recognized as the determinant of stem cell properties, there are also mounting evidence suggesting that an elaborate proteostasis network is crucial for stemness (Aguilo et al., 2015; Heintzman et al., 2009; Lee et al., 2017; San Jose et al., 2020; Yan et al., 2020; You et al., 2015). For example, defects in tRNA editing increase the accumulation of misfolded protein, which overwhelms the proteasomes in HSCs, and eventually impair its proliferation (San Jose et al., 2020). Likewise, chaperones also serve as significant determinants for stem cell pluripotency and differentiation (Bradley et al., 2012). In mESCs, the absence of Hsp60 and Hsp90 inhibits the expression of Oct4 by participating in the processing of *Oct4* mRNA (Bradley et al., 2012; Seo et al., 2018). Therefore, deciphering the proteostasis network and their interaction with the regulators of epigenome and transcriptome for maintaining the function of stem cells is worthy of further research.

Furthermore, defects in proteostasis lead to the dysfunction of somatic stem cells, and eventually result in impairment of organismal development and aging, which encourages studies of PQC in search of treatment of aging and aging-related diseases. Pathological conditions, environmental and metabolic stresses, and aging contribute to the production of aberrant proteins in addition to the normal and physiological sources of misfolded proteins (Haigis and Yankner, 2010). To maintain cellular protein homeostasis and restore viability, cells have developed a precise network to regulate and preserve the integrity of the proteome. Chaperones are essential for assisting protein folding and refolding of nascent and aberrant proteins. If the aberrant proteins cannot be refolded, they will be degraded through the ubiquitin-proteasome system or autophagy. Hence, deciphering in more detail the role of proteostasis during these events and the mechanisms underlying these changes will shed new light on the relationship between PQC and stem cell biology. As reviewed here, increasing evidence demonstrates that proteostasis plays a key role in the regulation of pluripotency (Lee et al., 2017). In addition, the regulators of PQC can also be core components in assisting the reprogramming process (Buckley et al., 2012).

Study of the regulatory network in proteostasis will also provide new aspects in understanding embryonic development, aging, and pathogenesis. For instance, misfolded proteins have been linked to many neurodegenerative diseases such as Huntington’s disease, Parkinson’s disease, and Alzheimer’s disease, in which aberrant protein aggregates overwhelm the cellular clearance machinery (Bosco et al., 2011; Finkbeiner, 2011; Schmidt and Finley, 2014; Selkoe, 2011). Recent researches suggested that protein misfolding is a key contributor to the progression of several diseases (Crunkhorn, 2015; Hartl et al., 2011) and loss of proteostasis has been implicated in stem cell aging (García-Prat et al., 2016). Failure of autophagy in physiologically aged satellite cells or genetic impairment of autophagy in young satellite cells results in senescence due to loss of proteostasis, accompanied by mitochondrial dysfunction and oxidative stress (García-Prat et al., 2016; Kapetanou et al., 2017). Further support for the link between PQC and longevity comes from studies demonstrating the activation of protein clearance mechanisms in longevity promoting pathways, which contribute to the amelioration of age-related diseases (Chondrogianni et al., 2000; Kapeta et al., 2010; Kenyon, 2010; Pérez et al., 2009). Further studies in mammals, especially in non-human primates, are required to unravel the potential links among PQC, aging, neurodegenerative disorders, and cancer. Nonetheless, increasing evidence supports the assertion that modulating PQC systems in stem cells will facilitate cell differentiation and reprogramming, interfere with cellular senescence, and sequentially reveal treatments with potential applications in clinical cell therapy. With this in mind, it will be crucial to develop drugs that activate the PQC systems to maintain proteostasis, potentially providing valuable therapeutic approaches for the treatment of aging and aging-related diseases.

## Abbreviations

PQC: Protein quality control
ESCs: Embryonic stem cells
ASCs: Adult stem cells
iPSCs: Induced pluripotent stem cells
PSCs: Pluripotent stem cells
ER: Endoplasmic reticulum
UPR: Unfolded protein response
UPS: Ubiquitin-proteasome system
HSPs: Heat shock proteins
NSCs: Neural stem cells
MSCs: Mesenchymal stem cells
NSPCs: Neural stem and progenitor cells
UPR^ER^: ER unfolded protein response
ATF6: Activated transcription factor 6
PERK: Double stranded RNA activated protein kinase-like ER kinase
IRE1α: inositol-requiring transmembrane kinase and endonuclease
HSCs: Hematopoietic stem cells
ROS: Reactive oxygen species
PAK2: p21-activated kinase 2
XBP1: X-box binding protein 1
UPR^MT^: Mitochondrial unfolded protein response
Nrf2: Nuclear factor erythroid 2-like 2
FBXW7: F box E3 ligase
ERAD: ER-associated degradation
EPAS1: Endothelial PAS domain protein 1
Pexophagy: Autophagy for peroxisomes
